# Molecular Epidemiology of Human Enterovirus 71 Strains and Recent Outbreaks in the Asia-Pacific Region: Comparative Analysis of the VP1 and VP4 Genes

**DOI:** 10.3201/eid0904.020395

**Published:** 2003-04

**Authors:** Mary Jane Cardosa, David Perera, Betty A. Brown, Doosung Cheon, Hung Ming Chan, Kwai Peng Chan, Haewol Cho, Peter McMinn

**Affiliations:** * Universiti Malaysia Sarawak, Sarawak, Malaysia; † Centers for Disease Control and Prevention, Atlanta, Georgia, USA; ‡ National Institute of Health, Seoul, Korea; § Singapore General Hospital, Singapore; ¶ Telethon Institute for Child Health Research, Perth, Western Australia, Australia

**Keywords:** human enterovirus 71, neurologic disease, hand, foot and mouth disease, research

## Abstract

This study provides a comprehensive overview of the molecular epidemiology of human enterovirus 71 (HEV71) in the Asia-Pacific region from 1997 through 2002. Phylogenetic analysis of the VP4 and VP1 genes of recent HEV71 strains indicates that several genogroups of the virus have been circulating in the Asia-Pacific region since 1997. The first of these recent outbreaks, described in Sarawak (Malaysian Borneo) in 1997, was caused by genogroup B3. This outbreak was followed by large outbreaks in Taiwan in 1998, caused by genogroup C2, and in Perth (Western Australia) in 1999, where viruses belonging to genogroups B3 and C2 cocirculated. Singapore, Taiwan, and Sarawak had HEV71 epidemics in 2000, caused predominantly by viruses belonging to genogroup B4; however, large numbers of fatalities were observed only in Taiwan. HEV71 was identified during an epidemic of hand, foot and mouth disease in Korea; that epidemic was found to be due to viruses constituting a new genogroup, C3.

Human enterovirus 71 (HEV71) was first isolated in 1969 (*1*) and is most often associated with outbreaks of the mild childhood exanthem, hand, foot and mouth disease (HFMD) (*2*). HEV71 is closely related to coxsackie virus A16 (CA16), the other major causative agent of HFMD, but unlike CA16, HEV71 is also associated with cases of acute neurologic disease including poliomyelitis-like paralysis, encephalitis, and aseptic meningitis (*3*,*4*). Although HEV71 outbreaks associated with small numbers of cases of severe neurologic disease were reported in the 1980s in Australia (*5*), Asia (*6*), and the United States (*4*), mortality associated with such outbreaks was low, unlike earlier outbreaks in Bulgaria in 1975 (*7*) and Hungary in 1978 (*8*). Twenty years later in 1997, deaths associated with epidemics of HEV71-associated HFMD in Sarawak, Malaysia (*9*), followed closely by outbreaks with high mortality in Taiwan in 1998 and 2000 (*10*,*11*), have raised considerable public concern about the virulence of this virus and the disease syndromes most recently attributed to it (*12*–*14*).

Several groups have attempted to describe the molecular epidemiology of HEV71 in the Asia-Pacific region, and the phylogenetic relationships of recent HEV71 strains to earlier strains from Asia and other parts of the world have been described (*15*–*19*). In the past 4 years, nine publications have presented comparative analyses of HEV71 strains based on several genome regions (Table 1). Taken together, more than 250 HEV71 strains isolated from the region between 1997 and 2002 have been analyzed by different groups. However, gaining a comprehensive insight into the phylogenetic relationships between all these HEV71 strains has been difficult because of the lack of a standardized methodology. The strategies used by each of these groups are summarized in Table 1.

**Table 1:** Summary of recent studies of human enterovirus 71 (HEV71) phylogeny

Reference	Gene/ region^a^	No. of nucleotides sequenced	No. of new strains (source, time)
AbuBakar et al., 1999 (20)	5′ UTR	440	13 (Malaysia, 1997)
Brown et al., 1999 (21)	VP1	891	113 (several countries, 1970–1998)
Shimizu et al., 1999 (15)	VP4/VP2	420	29 (Malaysia and Japan, 1997; Taiwan, 1998; 5 others (Bulgaria, Hungary, Japan, Taiwan, USA), 1973–1980)
Wang et al., 2000 (16)	5′ UTR	681	36 (Taiwan, 1998)
Shih et al., 2000 (17)	VP1	>500	16 (Taiwan, 1998)
Singh et al., 2000 (18)	VP1	341	20 (Singapore, Japan, and Peninsular Malaysia, 1997–1998)
Chu et al., 2001 (19)	VP4	207	20 (Taiwan, 1998); 3 (Taiwan, 1986)
McMinn et al., 2001 (22)	VP1	891	66 (Sarawak, Singapore, and Perth, 1997–2001)
Wang et al., 2002 (11)	5′ UTR VP1	648 841	26, 3, and 19 strains (Taiwan, 1998, 1999, and 2000) were sequenced in the 5′ UTR; 12, 2, and 19 strains (same 3 years) were sequenced in VP1

^a^UTR, untranslated region.

We sequenced the complete VP1 gene of 66 HEV71 isolates from Singapore, Sarawak (Malaysian Borneo), and Perth, Western Australia, from 1997 to early 2001 (*22*) and determined that these recent strains were from the genogroups B and C, based on the nomenclature of Brown et al. (*21*). In Sarawak in 1997, isolates from fatal and nonfatal cases all belonged to genogroup B, in a previously undescribed cluster that we have named B3 (*22*). Viruses belonging to genogroup B3 were also isolated in Singapore in 1998, and B3 was the genogroup most commonly identified for viruses isolated in Perth during 1999. However, HEV71 strains isolated from children with severe neurologic disease during the Perth epidemic belonged to genogroup C2 (*22*). Another previously undescribed genogroup B cluster (B4) was identified in Singapore in 1997 and continued to circulate there in 2000 (*23*) through 2002. Viruses from genogroup B4 were also identified as the primary cause of a large HFMD outbreak in Sarawak in 2000.

As several regions of the HEV71 genome have been used in phylogenetic studies, including the VP1 (*21*,*22*) and VP4 (*15*,*19*) genes and the 5′ untranslated region (5′ UTR) (*16*,*20*), undertaking a comprehensive analysis of the molecular epidemiology of recent HEV71 activity in the Asia-Pacific region has not been possible. To overcome this deficiency, we present here a comprehensive phylogenetic analysis of recent HEV71 strains, accomplished by examining both the VP1 and VP4 genes.

## Materials and Methods

### Viruses

All viruses used in this study were propagated in rhabdomyosarcoma cells before extraction of RNA. Thirty-nine representative HEV71 isolates from recent years in Sarawak, Singapore, Perth, and Korea, as well as 16 strains isolated in the United States from1972 to 1995, were propagated in rhabdomyosarcoma cells and subjected to nucleotide sequence analysis (see below). These strains were used to generate 12 new VP1 gene sequences and 55 new VP4 gene sequences (Appendix Table 1).

### Reverse Transcriptase–Polymerase Chain Reaction (RT-PCR)

RNA was extracted from infected rhabdomyosarcoma cell supernatants by using the High Pure viral nucleic acid kit (Roche Diagnostics GmbH, Mannheim, Germany). RT-PCR was performed as described (*22*), except that the annealing temperature for amplification of the VP4 gene was 50°C. Primer pairs 159/162 and 161/NP1A (*24*) were used for VP1 amplification of nucleotides (nt) 2385 to 2850 relative to BrCr; for VP4 amplification, the primers VP2-REV (5′ TTCCAATACCACCCCTTGGATGA 3′) and EVP-2 (*19*) were used to amplify nt 449 to 1192 relative to BrCr.

### Nucleotide Sequence Analysis

PCR products were purified from gels with the QIAquick gel extraction kit (QIAGEN Inc, Valencia, CA). Cycle sequencing was achieved with the primers 159/162 and 161/NP1A for VP1 and EVP-4 (*15*) and VP2-REV for VP4 by using the Big Dye Terminator Cycle Sequencing kit version 2.0 (Applied Biosystems, Foster City, CA). All sequences were determined for both strands by using the ABI377 automated DNA sequencer (Applied Biosystems).

### HEV71 Sequence Data Obtained from GenBank

The VP4 gene sequences of another 78 HEV71 strains obtained from GenBank ( Appendix Table 2) were included in this analysis, allowing the generation of dendrograms containing 128 HEV71 strains isolated from 1970 to 2002. These strains were isolated in the United States, Japan, Taiwan, Malaysia, Singapore, China, Bulgaria, Hungary, and the United Kingdom. In addition, 33 complete and 9 near-complete VP1 gene sequences of HEV71 strains retrieved from GenBank and used in this study are listed in  Appendix Table 2.

### Phylogenetic Analysis

Alignment of the VP1 and VP4 gene sequences was undertaken by using the ClustalW program (*25*). Dendrograms were constructed by using the neighbor-joining method with PHYLIP, version 3.5 (*26*) and drawn using TreeView (*27*). Bootstrap analysis with 1,000 pseudoreplicates was performed by using the program Seqboot (*28*). The CA16 strain G10 (*29*) was used as an outgroup for phylogenetic analysis of the VP4 sequence data, and the HEV71 prototype strain BrCr-CA-70 (*30*) was used as an outgroup for analysis of the VP1 sequence data. Historically Brown and co-workers (*21*) described genogroups as lineages of HEV71 distinguished by differing at least 15% in the VP1 gene. In this study we maintained the genogroups originally described and designated subgenogroups to aid in discussing evolving progeny viruses.

## Results

### Overview of HEV71 Phylogeny

**Figure 1:**
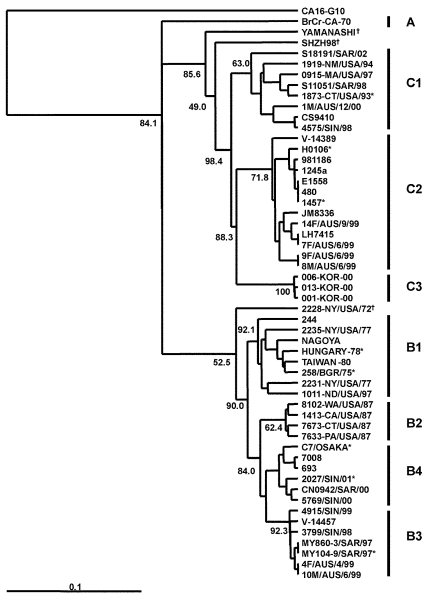
An overview of the genetic relationships of human enterovirus 71 (HEV71) strains isolated from 1970 through 2002. Dendrogram showing the genetic relationships among 53 HEV71 strains based on the alignment of the complete VP4 gene sequence (nucleotide positions 744–950). Details of the HEV71 strains included in the dendrogram are provided in Appendix Tables 1 and 2. Branch lengths are proportional to the number of nucleotide differences. The bootstrap values in 1,000 pseudoreplicates for major lineages within the dendrogram are shown as percentages. The marker denotes a measurement of relative phylogenetic distance. The VP4 nucleotide sequence of coxsackie virus A16 (CA16) (*29*) was used as an outgroup in the analysis. *Denotes HEV71 isolates from fatal cases; †denotes HEV71 strains falling outside existing genogroup boundaries.

**Figure 2:**
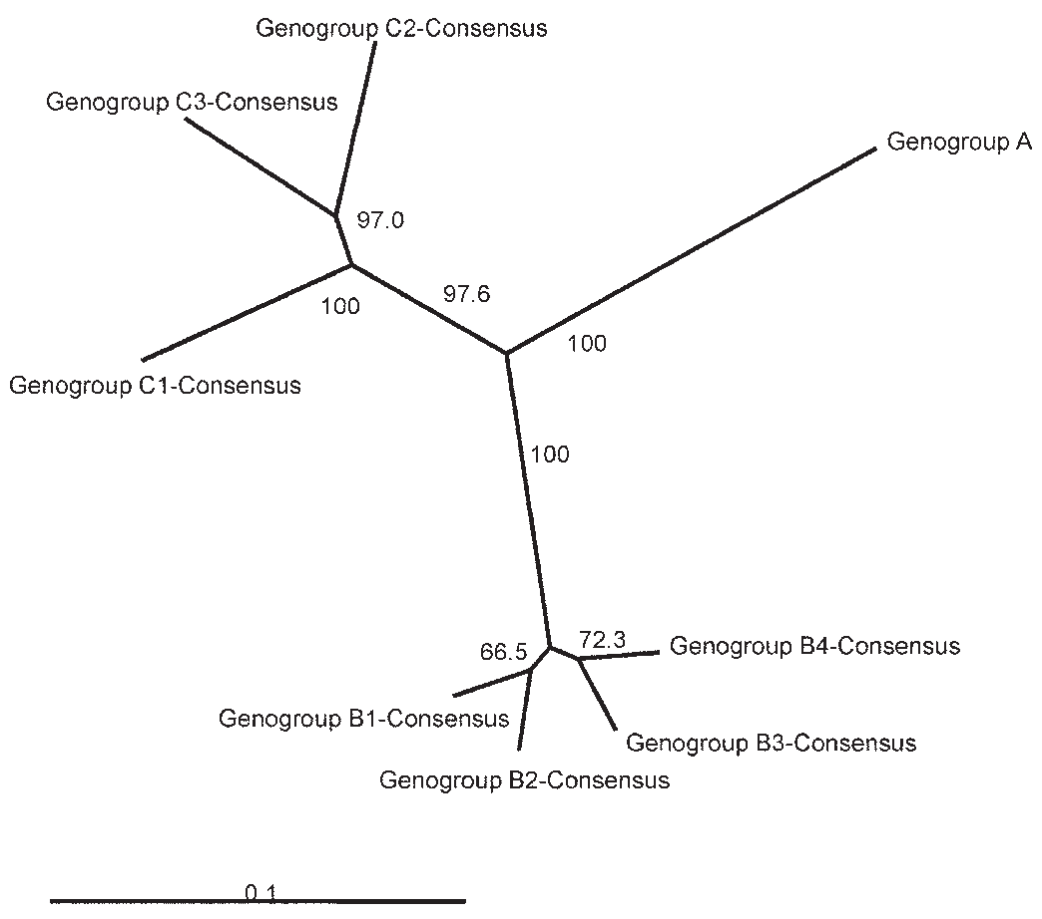
An overview of the genetic relationships of human enterovirus 71 (HEV71) strains isolated from 1970 through 2002. Unrooted cladogram shows the genogroup relationships of HEV71 based on an alignment of the partial VP4 gene (nucleotide positions 744–950) consensus sequences for genogroups B1, B2, B3, B4, C1, C2, and C3. The complete VP4 gene sequence of the prototype strain BrCr-CA-70 (30) was used as an outgroup in the analysis. The bootstrap values in 1,000 pseudoreplicates for major lineages within the dendrogram are shown as percentages. The marker denotes a measurement of relative phylogenetic distance.

Figure 1 presents an overview of the VP4-based phylogenetic tree, generated by including representative members of each of the genogroups A, B, and C. While there are a few outliers, this comprehensive VP4-based dendrogram accurately reproduces the genogroup clusters B1, B2, B3, B4, C1, and C2 (*21*,*22*) supported by the bootstrap values indicated. However, the Korean isolates form a distinct cluster in genogroup C with high bootstrap support, which we have designated genogroup C3. Selected HEV71 strains from the collection of the Centers for Disease Control and Prevention (CDC) were used to anchor the tree to maintain consistency of nomenclature between our VP4-based tree and the VP1-based tree published earlier by Brown et al. (*21*). The viruses in genogroups B and C share at least 78.3% nucleotide sequence identity. Within the B genogroup, the strains share at least 87.9% identity; the strains within the C genogroup share at least 84.5% identity. Overall, the C genogroup has greater diversity than the B genogroup. Within all subgenogroups, virus strains have >90% nucleotide sequence identity (Table 2). The divergence between genogroups A and B as well as A and C is 20% to 21%; the divergence between genogroups B and C is 16% to 25%. The strains in our collection from two of the more recently described genogroups B3 and C3 showed very high similarity to each other within the genogroup (>98% and 99% identity, respectively), while those within the B1 and C1 genogroups showed the widest divergence. The phylogenetic relationships between the consensus sequences of the different genogroups based on VP4 analysis are shown as an unrooted tree (Figure 2). This cladogram clearly illustrates that the C genogroup viruses are more divergent than the B genogroup viruses; however, because we might not have strains that are evenly distributed temporally and geographically, we were unable to determine if this difference in divergence means that genogroup B viruses have evolved more recently than the genogroup C viruses.

**Table 2:** VP4 nucleotide sequence identities within genogroups

Genogroup	% Identity
B1	91.3 to 100
B2	96.6 to 98.6
B3	98.1 to 100
B4	94.7 to 100
C1	90.3 to 100
C2	93.2 to 100
C3	99.0 to 100

### Genogroup B

**Figure 3:**
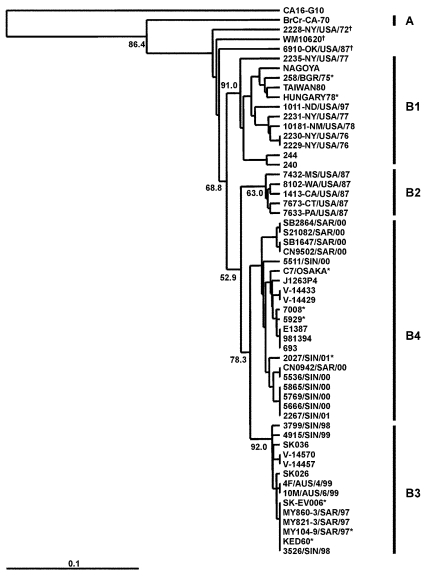
Phylogenetic relationships of human enterovirus 71 (HEV71) strains belonging to genogroup B (*21*). Dendrogram shows the genetic relationships among 56 HEV71 strains belonging to genogroup B, based on the alignment of the complete VP4 gene sequence (nucleotide positions 744-950). Details of the HEV71 strains included in the dendrogram are provided in Appendix Tables 1 and 2. Branch lengths are proportional to the number of nucleotide differences. The bootstrap values in 1,000 pseudoreplicates for major lineages within the dendrogram are shown as percentages. The marker denotes a measurement of relative phylogenetic distance. The VP4 nucleotide sequence of coxsackie virus A16 (CA16) (*29*) was used as an outgroup in the analysis. *Denotes HEV71 isolates from fatal cases; †Denotes HEV71 strains falling outside existing genogroup boundaries.

The four different genogroup B clusters described in our earlier VP1-based study (*20*) were accurately reproduced with VP4 gene sequences (Figure 3). This approach allows the inclusion of HEV71 strains from Taiwan and Japan published by Shimizu et al. (*15*) and Chu et al. (*19*). We show that, in the 1998 Taiwan epidemic, HEV71 strains belonging to genogroup B4 cocirculated in small numbers (e.g., 7008/98, 5929/98, and J1263P4). These strains are genetically similar to B genogroup viruses circulating in Japan during 1997 (V-14433, V-14429, C7/Osaka) but are distinct from the B3 genogroup of viruses circulating at the same time in Sarawak (e.g., MY104-9/SAR/97 and SK-EV006). Genogroup B4 strains have been circulating in the Asia-Pacific region at least since 1997, as these strains were isolated sporadically in Japan and Singapore in 1997 and in Taiwan during 1998, before the large outbreaks in 2000 in Singapore, Sarawak, and Taiwan associated with viruses from this genogroup. HEV71 strains of the B4 genogroup continued to be isolated in Singapore during 2001 and 2002.

**Figure 4:**
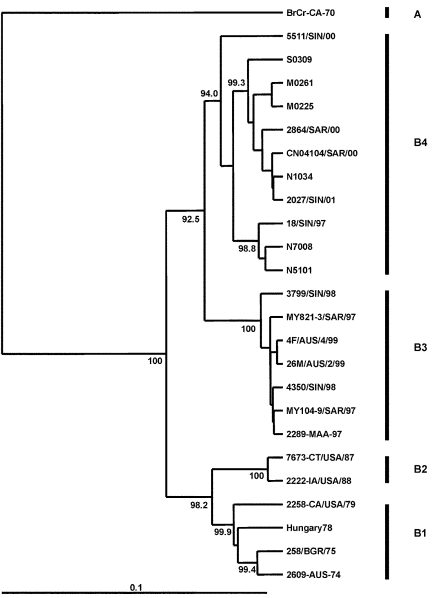
Phylogenetic relations of human enterovirus 71 (HEV71) strains belonging to genogroup B (*21*). Dendrogram shows the genetic relationships among 24 HEV71 strains belonging to genogroup B, based on the alignment of a partial VP1 (nucleotide positions 2442–3281) or complete VP1 (nucleotide positions 2442–3332) gene sequences. Details of the HEV71 strains included in the dendrogram are provided in Appendix Tables 1 and 2. Branch lengths are proportional to the number of nucleotide differences. The bootstrap values in 1,000 pseudoreplicates for major lineages within the dendrogram are shown as percentages. The marker denotes a measurement of relative phylogenetic distance. The VP1 nucleotide sequence of the prototype BrCr-CA-70 (*30*) was used as an outgroup in the analysis.

A Taiwanese group reported high death rates during an HEV71 outbreak in 2000 (*11*). Using phylogenetic analysis based on 840 nt of the VP1 gene (full length 891 nt), these investigators described a shift in genogroup from C in 1998 to B in 2000. To place the most recent Taiwanese strains in a regional context, we have included four representative genogroup B strains from the 2000 outbreak in Taiwan as well as two Taiwanese genogroup B strains from 1998 and 1999 in a limited VP1 gene-based dendrogram, together with representative strains from genogroups B1, B2, B3, and B4 (Figure 4). This analysis clearly identifies the Taiwan 2000 strains as belonging to genogroup B4. The temporal shift of B4 genogroup strains that occurred from one lineage in 1997 through 1999 (in Taiwan and Singapore) to a second lineage in 2000 through 2001 (in Taiwan, Singapore, and Sarawak) gave rise to large epidemics in these countries in 2000.

Another notable point that arises from VP4 gene-based phylogenetic analysis of genogroup B is that some older U.S. strains (2228-NY72 and 6910-OK87), which clearly belong within genogroup B1 in VP1-based analysis (*21*), do not lie within B1 in the VP4-based dendrogram (Figure 3). Moreover, in the United Kingdom a similar B genogroup “outlier” was isolated as recently as 1999. These outliers in the VP4-based analysis are very closely related to the B1 cluster, and this discrepancy probably results from the lower discriminating power obtained when using the VP4 gene for this analysis, as evidenced by lower bootstrap values at the major nodes.

### Genogroup C

**Figure 5:**
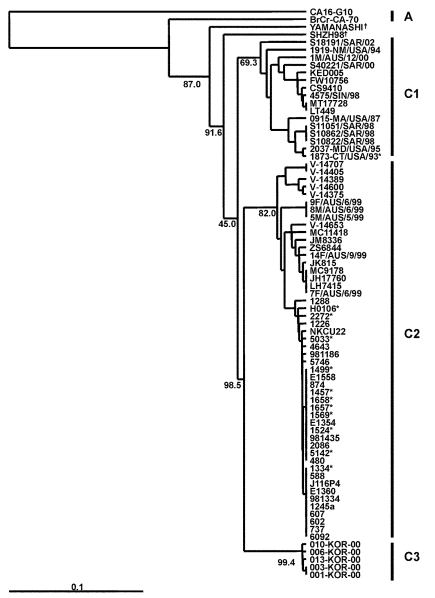
Phylogenetic relationships of human enterovirus 71 (HEV71) strains belonging to genogroup C (*21*). Dendrogram shows the genetic relationships among 74 HEV71 strains belonging to genogroup C, based on the alignment of the complete VP4 gene sequence (nucleotide positions 744–950). Details of the HEV71 strains included in the dendrogram are provided in Tables 2 and 3. Branch lengths are proportional to the number of nucleotide differences. The bootstrap values in 1,000 pseudoreplicates for major lineages within the dendrogram are shown as percentages. The marker denotes a measurement of relative phylogenetic distance. The VP4 nucleotide sequence of coxsackie virus A16 (*29*) was used as an outgroup in the analysis. *Denotes HEV71 isolates from fatal cases; †Denotes HEV71 strains falling outside existing genogroup boundaries.

**Figure 6:**
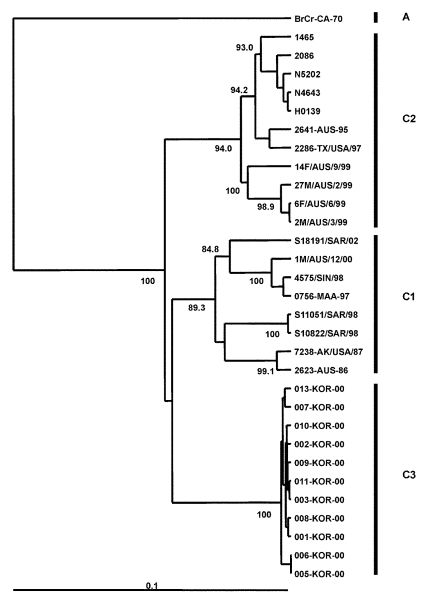
Phylogenetic relationships of human enterovirus 71 (HEV71) strains belonging to genogroup C (*21*). Dendrogram shows the genetic relationships among 30 HEV71 strains belonging to genogroup C, based on the alignment of a partial VP1 (nucleotide positions 2442–3281) or complete VP1 (nucleotide positions 2442–3332) gene sequences. Details of the HEV71 strains included in the dendrogram are provided in Tables 2 and 3. Branch lengths are proportional to the number of nucleotide differences. The bootstrap values in 1,000 pseudoreplicates for major lineages within the dendrogram are shown as percentages. The marker denotes a measurement of relative phylogenetic distance. The VP1 nucleotide sequence of the prototype BrCr-CA-70 (*30*) was used as an outgroup in the analysis.

Phylogenetic analysis that uses VP4 gene sequences also confirms previous observations that the major strains circulating in the Taiwan outbreak of 1998 were from genogroup C, more specifically C2, following the nomenclature of Brown et al. (*21*) (Figure 5). The Taiwanese isolates of 1998 clustered closely together in a lineage related to C2 strains isolated in Japan in the previous year and to those causing severe neurologic disease in Perth in 1999. This finding is supported by VP1-based analysis of Taiwanese strains from 1998 (Figure 6). We also located in GenBank a number of VP4 gene sequences of strains isolated in the United Kingdom between 1997 and 1999. These strains formed a cluster within genogroup C2, together with strains from Japan and Australia during 1997 and 1999, respectively. In our previous VP1-based study, the Australian C2 genogroup strains isolated during 1999 formed two distinct lineages (*22*), which are reproduced in the VP4 analysis. Similarly, Japanese isolates from 1997 form distinct lineages within genogroup C2.

An outbreak of HFMD with cases of aseptic meningitis and paralysis occurred in Korea during 2000; 11 strains of HEV71 from this outbreak are included in this study. All Korean strains formed a distinct cluster in genogroup C and showed 90% to 92% nt sequence identity with C1 and C2 subgroups and 79% to 82% identity with the different genogroup B strains. A dendrogram comparing the VP1 gene sequences of a limited number of HEV71 genogroup C strains with the 11 Korean isolates from 2000 and the single 2002 isolate from Sarawak is shown in Figure 6. This VP1-based dendrogram supports the VP4-based analysis, suggesting that the Korean HEV71 isolates form a distinct cluster, which we have designated C3. The Sarawak strain isolated in 2002 is from the C1 genogroup. Chu et al. (*19*) described the major genogroup circulating in Taiwan in 1998 as C3. However, because of the extent of variation in clusters, our previous analysis based on the VP1 gene designates the Taiwan outbreak strain as belonging to genogroup C2 (*22*); this current study establishes the designation C3 for the new Korean strains isolated during 2000.

The Japanese 1978 isolate “Yamanashi” is an outlier of the C genogroup. Twenty years later (1998), another C genogroup outlier was isolated in China (SHZH98). Both of these strains appear to be ancestral to C1, C2, and C3, raising the possibility that China may still have ancestral strains of HEV71 currently circulating.

## Discussion

Although several studies have attempted to describe the phylogenetic origins of HEV71 strains recently circulating in the Asia-Pacific region (*11*,*15*,*17*–*22*), no single study has included representative virus strains from all of the HEV71 epidemics recorded in the Asia-Pacific region since 1997 in a comprehensive analysis. We attempted to provide a more complete picture of the phylogenetic relationships between the HEV71 strains circulating in recent outbreaks and to place them in the context of strains isolated earlier and in other parts of the world since this virus was first described. The different genetic typing strategies and nomenclatures adopted by different groups have led to much confusion about the molecular epidemiology of recent HEV71 strains. Briefly summarizing these studies and linking the data presented into a single unifying picture are thus necessary.

Two very limited phylogenetic studies focused on short segments of the 5′ UTR. The first compared 13 strains of HEV71 isolated from fatal and nonfatal cases in Malaysia during 1997 (*20*) and showed that at least two lineages of this enterovirus circulated in Peninsular Malaysia during that period. HEV71 strains isolated from a pair of siblings, one with a fatal illness and one with uncomplicated HFMD, belonged to different genetic lineages. Wang et al. (*16*) also examined the 5′ UTR of 36 isolates from the large Taiwanese epidemic in 1998 and reported that most of the isolates clustered into a single genetic lineage, with two exceptions. Both of these studies are of limited value, not only because of the narrow focus on isolates from a single country in a single year, but also because the highly conserved 5′ UTR is not a suitable region upon which to base phylogenetic analysis of enteroviruses (*31*).

More recently, Wang et al*.* (*11*) examined the 5′ UTR and the VP1 gene of 58 Taiwan isolates isolated in 1998–2000 and showed that the major genogroup of HEV71 circulating in Taiwan changed from genogroup C in 1998 to genogroup B in 1999–2000. In this study, we reexamined nine HEV71 strains reported by Wang et al. and found that the major genogroup circulating during the 1998 Taiwan epidemic was C2, as we had shown previously (*22*). More importantly, our analysis shows that the major genogroup circulating in Taiwan changed from C2 in 1998 to B4 in 2000.

Two other phylogenetic analyses of HEV71 focus on the VP4 gene region. Shimizu et al. (*15*) examined a 420-nt region in the VP4/VP2 gene region of 16 isolates from Malaysia and Japan in 1997 and 13 isolates from Taiwan in 1998. These recent isolates were distributed into two major genogroups, which these researchers labeled genotypes A and B. (These genotype designations are not equivalent to genogroups A and B, originally described by Brown et al. [*21*]). Chu et al. (*19*) sequenced a 207-nt region of the VP4 gene of 23 Taiwanese strains. Twenty of the strains used in this study were isolated from fatal and nonfatal cases during the 1998 epidemic. Three strains isolated during the 1986 outbreak of HEV71-associated HFMD in Taiwan were included; this study confirmed that most 1998 Taiwanese strains were from a single genogroup, although several isolates were genetically distinct from the main group. We reexamined the VP4 genetic sequences from the HEV71 strains isolated in Taiwan during 1986 and found that they belonged to genogroup B1.

Use of the VP1 gene has been reported in four phylogenetic studies of HEV71 strains derived from recent HFMD epidemics in the Asia-Pacific region. Two of these studies appear to be of limited value, having analyzed only short VP1 gene sequences and limited numbers of HEV71 isolates within restricted geographic areas. Singh et al. (*18*) presented a dendrogram based on a 341-nt region of the VP1 gene and concluded that several recent Southeast Asian strains belonged to two completely new genotypes. However, this finding appears to be flawed, as closer examination of the sequences used in construction of the dendrogram shows that they include a mixture of partial VP1 gene sequences and 5′ UTR sequences from (occasionally) the same strain. Shih et al. (*17*) examined partial VP1 gene sequences from 16 isolates from fatal and nonfatal cases in Taiwan during 1998; Shih’s study provided further confirmation that most isolates from the 1998 Taiwan outbreak belonged to a single genogroup. Shih et al. (*17*) described this predominant genogroup as “genotype B” and the minor genogroup as “genotype C” but labeled the isolates in their dendrograms in the reverse order. If one assumes that the dendrogram labeling is correct, their findings are consistent with the nomenclature of Brown et al. (*21*).

In our study, we used all available published data in addition to new sequence data from the VP4 gene of 51 recent strains and the VP1 gene of 11 Korean strains and 1 Sarawak strain of HEV71 to provide a comprehensive picture of the molecular epidemiology of HEV71 in the Asia-Pacific region since 1997. Our study shows that the first recent HEV71 outbreak recognized in the region occurred in Sarawak in 1997 and was associated with the previously undescribed genogroup B3. Viruses belonging to this genogroup were also circulating in Singapore and Japan in 1997, continued to circulate in Singapore in 1998, and were the primary cause of the epidemic in Western Australia in 1999. Since 1999, genogroup B3 viruses have not been identified anywhere within the region. In 1998, viruses of the C2 genogroup were the primary cause of the Taiwanese epidemic; strains from a distinct lineage within the same genogroup also circulated in Western Australia during 1999. Viruses belonging to the C1 genogroup appear to have undergone low-level endemic circulation in Malaysia, Singapore, and Perth between 1997 and 2002 and have not been associated with large-scale epidemics to date.

Our analysis shows that a great diversity of HEV71 strains circulate in the region and elsewhere and that no particular genogroup is specifically associated with severe disease. Since we were hampered by the fact that many studies failed to provide an accurate diagnosis for the cause of death in fatal cases of HEV71 infection, we suggest that identification of cases of encephalitis, poliomyelitis-like paralysis, or both is likely to provide a more accurate endpoint in the determination of disease severity associated with particular HEV71 genogroups. At this time, insufficient published data are available relating to cause of death to perform this analysis. Currently, the best available data on disease severity are from our previous study (*20*), in which viruses isolated from children with severe neurologic disease belonged exclusively to genogroup C2 and possessed a unique amino acid substitution in VP1 at position 170(A→V).

Genogroup B4 strains were isolated occasionally in Peninsular Malaysia, Singapore, and Taiwan from 1997 to 1999. In 2000, viruses from this genogroup caused large epidemics in Sarawak, Singapore, and Taiwan; however, in Korea, an outbreak of HFMD was caused by the new C3 genogroup. Table 3 shows the major HEV71 genotypes circulating in countries within the region since 1997. Clearly, HEV71 is a major emerging virus in the region. Because of this virus’ potential for causing severe neurologic disease, we need to understand the factors that have led to the spread of this virus and the genetic factors that contribute to its neurovirulence and epidemic potential.

**Table 3:** Summary of human enterovirus 71 (HEV71) genotypes circulating in the Asia-Pacific region since 1997^a^

	1997	1998	1999	2000	2001	2002
Singapore	B3, B4	B3, C1	B3	**B4**	B4	C1, B4
Sarawak, Malaysia	**B3**	C1	No HEV71	**B4**, C1	No HEV71	C1
Perth, Australia	-	-	**B3, C2**	C1	No HEV71	No HEV71
Japan	B3, B4, C2	-	-	-	-	-
Taiwan	-	**C2**, B4	B4	**B4**	-	-
Korea	-	-	-	**C3**	No HEV71	No HEV71

^a^Boldface type indicates genogroups causing large outbreaks; –, no data available; No HEV71, no HEV71 identified despite active surveillance.

In conclusion, VP1 and VP4 gene sequences both provide similar phylogenetic information, but the higher bootstrap values seen in the VP1 dendrograms provide greater confidence, particularly when elucidating new genotypes. Thus, the use of the shorter VP4 gene may be helpful for HEV71 surveillance, but the VP1 gene should still be used for molecular epidemiologic research and for confirming data obtained with VP4-based analysis. Virus identification and classification have been reliant on antigenic methods for serotypic identification, and VP1 gene sequence data have been shown to infer serotype (*32*). Furthermore, the protein encoded by the VP1 gene is the most exposed and immunodominant of the capsid proteins (*33*) and is likely to give the most useful information in molecular epidemiologic investigations, unlike untranslated sequences or genes, which encode products not found exposed on the virion surface.

## Supplementary Material

Appendix Table 1 Human enterovirus 71 (HEV71) strains sequenced for this study

Appendix Table 2 Human enterovirus 71 (HEV71) sequences obtained from GenBank
