# Supplementary material for: Molecular Epidemiology of Human Enterovirus 71 Strains and Recent Outbreaks in the Asia-Pacific Region: Comparative Analysis of the VP1 and VP4 Genes
Source: Emerg Infect Dis. 2003 Apr;9(4):462–8. doi: 10.3201/eid0904.020395 (PMC2957976; doi:10.3201/eid0904.020395)
Supplement: Appendix Table 1 — Human enterovirus 71 (HEV71) strains sequenced for this study [file 02-0395_appT1-s1.pdf]

Appendix Table 1. Human enterovirus 71 (HEV71) strains sequenced for this study

Identity (strain/origin[a]/yr)	Accession no.[b]	Genogroup based on VP1	Genogroup based on VP4	Clinical syndrome[c]
2228-NY72/USA/72	AY123949	B1	B outlier	NA
2229-NY76/USA/76	AY123950	B1	B1	NA
2230-NY76/USA/76	AY123951	B1	B1	NA
2231-NY77/USA/77	AY123952	B1	B1	NA
2235-NY77/USA/77	AY123953	B1	B1	NA
10181-NM78/USA/78	AY123944	B1	B1	NA
1011-ND79/USA/79	AY123943	B1	B1	NA
6910-OK87/USA/87	AY123954	B1	B outlier	Rash
1413-CA87/USA/87	AY123945	B2	B2	Paralysis
7633-PA87/USA/87	AY123955	B2	B2	Gastroenteritis
7673-CT87/USA/87	AY123956	B2	B2	NA
8102-WA87/USA/87	AY123957	B2	B2	Meningitis
MY104-9/SAR/97	AY125997	B3	B3	Acute cardiogenic shock, fatal
MY821-3/SAR/97	AY125995	B3	B3	Meningitis
MY860-3/SAR/97	AY125996	B3	B3	HFMD
3526/SIN/98	AY125984	B3	B3	Encephalitis
3799/SIN/98	AY125985	B3	B3	HFMD
4915/SIN/99	AY125987	B3	B3	Meningitis
4F/AUS/4/99	AY126008	B3	B3	Guillain-Barré syndrome
10M/AUS/6/99	AY126007	B3	B3	HFMD
SB1647/SAR/00	AY126004	B4	B4	Meningitis
SB2864/SAR/00	AY126006	B4	B4	Poliomyelitis-like paralysis
CN0942/SAR/00	AY126001	B4	B4	HFMD
CN9502/SAR/00	AY126002	B4	B4	HFMD
S21082/SAR/00	AY126005	B4	B4	HFMD
5511/SIN/00	AY125988	B4	B4	HFMD
5536/SIN/00	AY125991	B4	B4	HFMD
5769/SIN/00	AY125992	B4	B4	Encephalitis
2027/SIN/01	AY125993	B4	B4	Encephalitis, fatal
2267/SIN/01	AY125994	B4	B4	Febrile illness
0915-MA87/USA/87	AY123942	C1	C1	Meningitis
1873-CT94/USA/94	AY123946	C1	C1	Fatality
1919-NM94/USA/94	AY123947	C1	C1	Rash
2037-MD95/USA/95	AY123948	C1	C1	NA
4575/SIN/98	AY125986	C1	C1	HFMD
S10822/SAR/98	AY125998	C1	C1	HFMD
S10862/SAR/98	AY125999	C1	C1	HFMD
S11051/SAR/98	AY126000	C1	C1	HFMD
S40221/SAR/00	AY126003	C1	C1	HFMD
S18191/SAR/02	AY189153, AY189154 (VP1)	C1	C1	HFMD
1M/AUS/12/00	AY126014	C1	C1	HFMD
5M/AUS/5/99	AY126009	C2	C2	Meningitis

8M/AUS/6/99	AY126012	C2	C2	Myelitis
9F/AUS/6/99	AY126013	C2	C2	Cerebellar ataxia
7F/AUS/6/99	AY126010	C2	C2	Meningitis
14F/AUS/9/99	AY126011	C2	C2	Benign intracranial hypertension
KOR-EV71-01/KOR/00	AY125966 (VP1), AF525220	C3	C3	HFMD
KOR-EV71-03/KOR/00	AY125968 (VP1), AF525221	C3	C3	HFMD
KOR-EV71-06/KOR/00	AY125970 (VP1), AF525222	C3	C3	Aseptic meningitis
KOR-EV71-10/KOR/00	AY125974 (VP1), AF525223	C3	C3	HFMD/Herpangina
KOR-EV71-13/KOR/00	AY125976 (VP1), AF525224	C3	C3	Paralysis
KOR-EV71-02/KOR/00	AY125967 (VP1)	C3	-	HFMD
KOR-EV71-05/KOR/00	AY125969 (VP1)	C3	-	Aseptic meningitis
KOR-EV71-07/KOR/00	AY125971 (VP1)	C3	-	Aseptic meningitis
KOR-EV71-08/KOR/00	AY125972 (VP1)	C3	-	Aseptic meningitis
KOR-EV71-09/KOR/00	AY125973 (VP1)	C3	-	HFMD/Herpangina
KOR-EV71-11/KOR/00	AY125975 (VP1)	C3	-	HFMD/Herpangina

[a]Place of origin is abbreviated as follows: AUS, Australia; KOR, Korea; SAR, Sarawak, Malaysian Borneo; SIN, Singapore; USA, United States of America.

[b]Unless otherwise indicated, accession number refers to a VP4 sequence.

[c]NA, not available; HFMD, hand, foot and mouth disease.
