# Supplementary material for: Molecular Epidemiology of Human Enterovirus 71 Strains and Recent Outbreaks in the Asia-Pacific Region: Comparative Analysis of the VP1 and VP4 Genes
Source: Emerg Infect Dis. 2003 Apr;9(4):462–8. doi: 10.3201/eid0904.020395 (PMC2957976; doi:10.3201/eid0904.020395)
Supplement: Appendix Table 2 — Human enterovirus 71 (HEV71) sequences obtained from GenBank [file 02-0395_appT2-s2.pdf]

Appendix Table 2. Human enterovirus 71 (HEV71) sequences obtained from GenBank

Identity strain(origin[a]/yr)[b]	Accession no.	Gene(s) used in analysis
CA16-G10(SAF/51)	NC001612	VP4
BrCr-CA-70(USA/70)	U22521	VP1 and VP4
MS/7423/87(USA/87)	U22522	VP4
SHZH98(CHN/98)	AF302996	VP4
Nagoya(JPN/73)	AB051301	VP4
258(BGR/75)	AB051302	VP1 and VP4
Hungary(HGR/78)	AB051303	VP1 and VP4
Yamanashi(JPN/78)	AB051305	VP4
Taiwan 80(TWN/80)	AB051304	VP4
V-14375(JPN/97)	AB051319	VP4
V-14389(JPN/97)	AB051320	VP4
V-14405(JPN/97)	AB051321	VP4
V-14433(JPN/97)	AB051322	VP4
V-14429(JPN/97)	AB051323	VP4
V-14570(JPN/97)	AB051324	VP4
V-14600(JPN/97)	AB051325	VP4
V-14653(JPN/97)	AB051326	VP4
V-14707(JPN/97)	AB051327	VP4
C7/Osaka(JPN/97)	AB051328	VP4
V-14457(JPN/97)	AB051329	VP4
SK-EV006(SAR/97)	AB051331	VP4
SK026(MAL/97)	AB051332	VP4
SK036(MAL/97)	AB051333	VP4
KED005(MAL/97)	AB051334	VP4
KED60(MAL/97)	AB051335	VP4
1334(TWN/98)	AB051306	VP4
1457(TWN/98)	AB051307	VP4
1524(TWN/98)	AB051308	VP4
981394(TWN/98)	AB051309	VP4
981186(TWN/98)	AB051310	VP4
981334(TWN/98)	AB051311	VP4
981435(TWN/98)	AB051312	VP4
E1387(TWN/98)	AB051313	VP4
E1558(TWN/98)	AB051314	VP4
J1263P4(TWN/98)	AB051315	VP4
J1166P4(TWN/98)	AB051316	VP4
E1354(TWN/98)	AB051317	VP4
E1360(TWN/98)	AB051318	VP4
TW/2272/98(TWN/98)	AF119795	VP4
TW/2086/98(TWN/98)	AF119796	VP4
240/86(TWN/86)	AB037267	VP4
244/86(TWN/86)	AB037268	VP4
5033/98(TWN/98)	AB037250	VP4

5142/98(TWN/98)	AB037251	VP4
H0106/98(TWN/98)	AB037252	VP4
1657/98(TWN/98)	AB037253	VP4
1658/98(TWN/98)	AB037254	VP4
1569/98(TWN/98)	AB037255	VP4
1499/98(TWN/98)	AB037256	VP4
480/98(TWN/98)	AB037257	VP4
874/98(TWN/98)	AB037258	VP4
737/98(TWN/98)	AB037259	VP4
607/98(TWN/98)	AB037260	VP4
602/98(TWN/98)	AB037261	VP4
588/98(TWN/98)	AB037262	VP4
1226/98(TWN/98)	AB037263	VP4
1288/98(TWN/98)	AB037264	VP4
693/98(TWN/98)	AB037266	VP4
5929/98(TWN/98)	AB046739	VP4
7008/98(TWN/98)	AB046740	VP4
1245a/98/tw(TWN/98)	AF176044	VP4
Tainan/5746/98(TWN/98)	AF304457	VP4
Tainan/4643/98(TWN/98)	AF304458	VP4
Tainan/6092/98(TWN/98)	AF304459	VP4
NCKU9822(TWN/98)	AF136379	VP4
ZS6844/96(UK/96)	AJ300756	VP4
JM8336/97(UK/97)	AJ300761	VP4
JH17760/98(UK/98)	AJ300760	VP4
MT17728/98(UK/98)	AJ300764	VP4
LH7415/99(UK/99)	AJ300753	VP4
JK815/99(UK/99)	AJ300754	VP4
WM10620/99(UK/99)	AJ300755	VP4
CS9410/99(UK/99)	AJ300758	VP4
FW10756/99(UK/99)	AJ300759	VP4
MC11418/99(UK/99)	AJ300762	VP4
MC9178/99(UK/99)	AJ300763	VP4
LT449/00(UK/00)	AJ300757	VP4
5865/sin/000009(SIN/00)	AF316321	VP4
5666/sin/002209(SIN/00)	AF352027	VP4
S0309-TW-00(TWN/00)	AY055176	VP1
M0267-TW-00(TWN/00)	AY055184	VP1
M0225-TW-00(TWN/00)	AY055190	VP1
N1034-TW-00(TWN/00)	AY044181	VP1
N7008-TW-99(TWN/99)	AY055201	VP1
N5101-TW-98(TWN/98)	AY055174	VP1
N5202-TW-98(TWN/98)	AY055200	VP1
N4643-TW-98(TWN/98)	AY055194	VP1
H0139-TWN-98(TWN/98)	AY055193	VP1
7673-CT-87(USA/87)	AF009535	VP1
MY104-9/SAR/97	AF376072	VP1
MY821-3/SAR/97	AF376077	VP1

3799/SIN/98	AF376117	VP1
4F/AUS/4/99	AF376105	VP1
SB2864/SAR/00	AF376066	VP1
5511/SIN/00	AF376121	VP1
2027/SIN/01	AF376111	VP1
4575/SIN/98	AF376120	VP1
S10822/SAR/98	AF376079	VP1
S11051/SAR/98	AF376081	VP1
1M/AUS/12/00	AF376098	VP1
14F/AUS/9/99	AF376091	VP1
TW/2086/98(TWN/98)	AF119796	VP1
CN04104/SAR/00	AF376067	VP1
26M/AUS/2/99	AF376101	VP1
18/Sin/97(SIN/97)	AF251359	VP1
4350/SIN/98	AF376119	VP1
2289-MAA-97(SAR/97)	AF135914	VP1
2222-IA-88(USA/88)	AF009540	VP1
2258-CA-79(USA/79)	AF135880	VP1
2609-AUS-74(AUS/74)	AF135886	VP1
TW/1465/98(TWN/98)	AF116814	VP1
2641-AUS-95(AUS/95)	AF135947	VP1
2286-TX-97(USA/97)	AF135941	VP1
27M/AUS/2/99	AF376102	VP1
6F/AUS/6/99	AF376107	VP1
2M/AUS/3/99	AF376103	VP1
7238-AK-87(USA/87)	AF135952	VP1
2623-AUS-86(AUS/86)	AF135945	VP1
0756-MAA-97(MAL/97)	AF135935	VP1

[a]Place of origin is abbreviated as follows: AUS, Australia; BGR, Bulgaria; CHN, China; HGR, Hungary; JPN, Japan; KOR, Korea; MAL, Peninsular Malaysia; SAF, South Africa; SAR, Sarawak, Malaysian Borneo; SIN, Singapore; TWN, Taiwan; U.K., United Kingdom; USA, United States of America.

[b]All sequences retrieved from GenBank are referred to by the strain name recorded in the GenBank record followed by place of origin and year of isolation in brackets. Strains sequenced during this collaborative project have been deposited with the information on origin and year already included in the strain name following the same nomenclature as in Appendix Table 1.
